# Multiple imputation of missing data under missing at random: including a collider as an auxiliary variable in the imputation model can induce bias

**DOI:** 10.3389/fepid.2023.1237447

**Published:** 2023-09-15

**Authors:** Elinor Curnow, Kate Tilling, Jon E. Heron, Rosie P. Cornish, James R. Carpenter

**Affiliations:** 1 Department of Population Health Sciences, Bristol Medical School, University of Bristol, Bristol, United Kingdom; 2 Medical Research Council Integrative Epidemiology Unit at the University of Bristol, University of Bristol, Bristol, United Kingdom; 3 department of Medical Statistics, London School of Hygiene and Tropical Medicine, University of London, London, United Kingdom; 4 Medical Research Council Clinical Trials Unit at University College London, University of London, London, United Kingdom

**Keywords:** missing data, multiple imputation, collider bias, auxiliary variable, ALSPAC

## Abstract

Epidemiological studies often have missing data, which are commonly handled by multiple imputation (MI). In MI, in addition to those required for the substantive analysis, imputation models often include other variables (“auxiliary variables”). Auxiliary variables that predict the partially observed variables can reduce the standard error (SE) of the MI estimator and, if they also predict the probability that data are missing, reduce bias due to data being missing not at random. However, guidance for choosing auxiliary variables is lacking. We examine the consequences of a poorly chosen auxiliary variable: if it shares a common cause with the partially observed variable and the probability that it is missing (i.e., it is a “collider”), its inclusion can induce bias in the MI estimator and may increase the SE. We quantify, both algebraically and by simulation, the magnitude of bias and SE when either the exposure or outcome is incomplete. When the substantive analysis outcome is partially observed, the bias can be substantial, relative to the magnitude of the exposure coefficient. In settings in which a complete records analysis is valid, the bias is smaller when the exposure is partially observed. However, bias can be larger if the outcome also causes missingness in the exposure. When using MI, it is important to examine, through a combination of data exploration and considering plausible casual diagrams and missingness mechanisms, whether potential auxiliary variables are colliders.

## 1 Introduction

Missing data are ubiquitous in health and social research, with multiple imputation (MI) a commonly used, general and flexible method for analysing partially observed datasets (1). When imputation models are appropriately specified, MI gives valid inferences if data are missing completely at random (MCAR) or missing at random (MAR), conditional on the observed data, but not (unless additional information is available) if data are missing not at random (MNAR) (Table 1). In MI, in addition to the variables used in the analysis model, imputation models often include auxiliary variables (Table 1). Auxiliary variables have two main functions: (1) to improve the predictive ability of the imputation model, over and above the information recovered via the analysis model variables, thus increasing precision (3); and (2) to reduce bias due to data being MNAR (this is sometimes described as “making the MAR assumption more plausible”) (4). However, previous studies have shown that inclusion of auxiliary variables that are only weakly correlated with the partially observed variable, conditional on the remaining imputation model variables, can increase the standard error (SE) of the MI estimate (3, 5). In this paper, we highlight another, little known, consequence of incorrect choice of auxiliary variable: inclusion of an auxiliary variable that shares a common cause with the partially observed variable and its missingness (in causal inference, such a variable is referred to as a “collider” (6)) can lead to biased MI estimates by inducing a MNAR mechanism. We also demonstrate that inclusion of a collider in the imputation model may also increase the SE, despite the collider being (conditionally) predictive of the missing data. The consequences of including a collider in the imputation model were discussed in principle by Thoemmes and Rose (7). Here, we quantify the bias and SE of the MI estimator based on a collider. We expand the scenarios discussed by Thoemmes and Rose, considering settings in which the (continuous or binary) partially observed variable is either the analysis model outcome or the exposure. We further illustrate our results using simulation and real data examples. All analyses were conducted using Stata version 17.0 (StataCorp LLC, College Station, TX, USA). The Stata code used to perform the simulation studies is included in Supplementary Material, Section S8. The Stata code used to perform the real data analysis is included in Supplementary Material, Section S9.

## 2 Bias and SE of the MI estimator including a collider in the imputation model when a continuous outcome is partially observed

### 2.1 Model set-up

**Figure 1:**
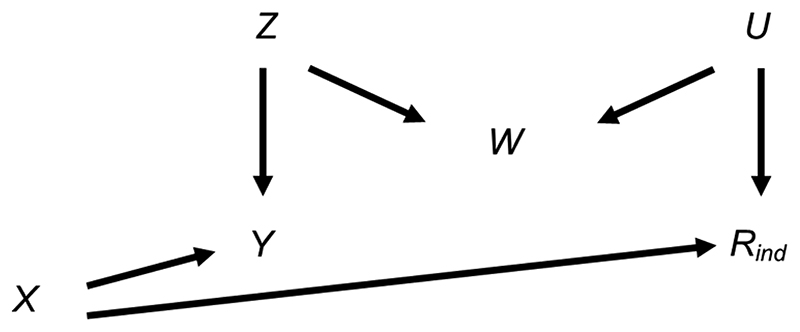
Directed acyclic graph depicting the relationship between outcome *Y*, exposure *X*, missingness indicator *R*_ind_ and potential auxiliary variables *Z*, *W* and *U.* Lines indicate related variables, with arrows indicating the direction of the relationship;absent lines represent variables with no direct causal relation.

We first consider the setting depicted in the causal diagram [or directed acyclic graph (DAG)] in Figure 1 (lines indicate related variables, with arrows indicating the direction of the relationship; absent lines represent variables with no direct causal relation). We examine the bias and SE of the MI estimator in detail in this simplified setting, to give insights into the more complex settings that typically occur in epidemiological practice. Suppose, for example, Figure 1 depicts the relationship between a child’s body mass index (BMI) at the age of 7 years (our continuous outcome, denoted by *Y*) and maternal education (our exposure, denoted by *X*), with *β_YX_* denoting the parameter of interest. Further suppose that the BMI at age 7 years is partially observed (with binary variable *R*_ind_ indicating whether BMI at age 7 years is observed, such that *R*_ind_ =1 if BMI is observed, and 0 otherwise), maternal education is fully observed, and that there are only two fully observed candidate auxiliary variables available for use in the imputation model for BMI at age 7 years: pregnancy size (singleton vs. twin birth, denoted by *Z* in Figure 1); and child’s birth weight (denoted by W in Figure 1) —noting that in reality there will be many other measured variables related to those discussed here. Here, we assume that birth weight is related to both BMI at age 7 years (via pregnancy size) and its missingness (via some unmeasured variable(s), depicted as *U* in Figure 1). Since birth weight shares a common cause with both BMI at age 7 years and its missingness, we say that birth weight is a “collider” of BMI at age 7 years and its missingness. Note that the plausibility of our assumptions is discussed in the real data analysis, in Section 3.

We further assume that we know (having considered the DAG) the “substantive model” we would fit to address our scientific question if there were no missing data. In this case, this is simply the regression of BMI at age 7 years on maternal education, because the other variables depicted in the DAG do not confound the relationship between BMI at age 7 years and maternal education.

Since *R*_ind_ is unrelated to BMI at age 7 years conditional on maternal education, both complete records analysis (CRA) and MI using maternal education as the predictor in the imputation model for BMI at age 7 years are valid analysis strategies (8) and will yield unbiased estimates given correctly specified models. Note, as per current guidelines (9), the imputation model should always include the other analysis model variable(s), i.e., maternal education. However, MI using just maternal education will recover no additional information compared to CRA (10). Therefore, we may wish to include auxiliary variables in our imputation model (i.e., either pregnancy size, birth weight or both) to improve the precision of our estimate of *β_YX_*. Our aim is to choose the most appropriate set of predictors to include in the imputation model for BMI at age 7 years, choosing between the following: (1) maternal education; (2) maternal education and pregnancy size; (3) maternal education and birth weight; or (4) maternal education, pregnancy size and birth weight. In addition to set (1), already discussed, sets (2) and (4) (i.e., including pregnancy size, or birth weight and pregnancy size, in the imputation model as well as maternal education) are valid analysis strategies. However, since birth weight is a collider, set (3) (including maternal education and birth weight but not pregnancy size in the imputation model) will induce bias in the MI estimator. In causal inference, this type of bias is often referred to as “M-bias” (11), due to the “M” shape of the causal pathways, as shown in Figure 1. Note that bias will be induced, regardless of the distribution of the variables and/or the form of their relationships (e.g., whether these are linear or non-linear), because the rules of DAGs that we have applied here do not make any distributional assumptions. However, the magnitude of the induced bias and the SE of the MI estimator will depend on the distributions and forms of relationships of all the variables. In the following sections, we explore the consequences, in terms of bias and precision, of choosing set (3) as predictors in the imputation model, deriving theoretical results for bias and SE in the specific setting in which the analysis outcome is continuous and all variables are normally distributed, with linear associations.

### 2.2 Bias in the MI estimator when including a collider in the imputation model for a continuous outcome as the proportion of missing data increases

We first provide a general expression for the bias of the MI estimator when including a collider in the imputation model for a continuous outcome (i.e., in terms of the variables in Figure 1, using *X* and *W*, but not *Z*, as predictors in the imputation model for *Y*). A detailed explanation of this result is included in the Supplementary Material (Section S1). The main argument is described below.

We assume that *Y*, *X*, *Z*, *U* and W are normally distributed, and *R*_ind_ is defined as follows: there exists a normally distributed variable *R* with mean *μ_R_* and variance *V_R_*, such that $P(R_{\text{ind}}=1)=P(R\leq r)=\text{Φ(}\frac{r-\mu_{R}}{\sqrt{V_{R}}}),$ where **Φ** denotes the cumulative distribution function of the standard normal distribution. Furthermore, we assume that each of *Y*, *W* and *R* is a linear combination of the variables causing it plus an error term (with *X*, *Z* and *U* having no direct causes), with no interactions, all errors uncorrelated, no model mis-specification and no measurement error. Finally, we assume an ordinary least squares (OLS) estimator is used to obtain estimates in both analysis and imputation models.

We consider the situation in which MI is performed by replacing missing values of Y with draws from a linear regression model [note this is the default method for continuous variables when using *mi impute* in Stata (12) or *proc mi* in SAS (13), although predictive mean matching (14) is the default method when using mice in R (15)]. As described above, we assume both *X* and *W* are included as predictors in the imputation model for *Y*, i.e., the imputation model is of the form: *E*(*Y*) = *α*_0_ + *α_1_X* + *α_2_W*, where E(.) denotes the expected value. Following the argument of Carpenter and Kenward (5) and noting, implicit from Figure 1, that *β_YX_* conditional on *W(ß_YX_|_W_*) is equivalent to *β_YX_* in our scenario, the MI estimator of *β_YX_* (denoted by $\beta_{YX}^{\text{MI}}$) equals the regression parameter for *X* from the imputation model for *Y* based on records with observed values of *Y* (we denote this parameter by $\alpha_{1}^{\text{OBS}}$). Hence, the MI estimator is unbiased only if $\alpha_{1}^{\text{OBS}}$ is unbiased.

In general (see Supplementary Material Section S1 for further explanation of this result), the bias of the MI estimator is bounded as follows: 0 ≤ bias ≤ |*ß*_YX|WR_ – *ß_YX_*|. If there are no missing values of *Y*, the MI estimator is unbiased. As the probability that *Y* is missing (i.e., *P*(*R*_ind_ = 0), denoted by π_0_) increases, the magnitude of bias of the MI estimator increases. In the hypothetical situation in which all values are missing, bias takes its maximum value of *β_YX_|_WR_ – β_YX_*|.

### 2.3 Standard error of the MI estimator when including a collider in the imputation model for a continuous outcome as the proportion of missing data increases

Here, we provide general formulas for quantifying the SE of the MI estimator when including a collider in the imputation model, additionally comparing this to the SE of the CRA estimator.

The SE of the MI estimator when including collider *W* in the imputation model, $\text{SE}(\beta_{YX}^{\text{MI}}),$ will always be greater than the SE of the imputation model coefficient $\alpha_{1}^{\text{OBS}},$ with $\text{SE}(\alpha_{1}^{\text{OBS}}),$ as defined above, tending towards $\text{SE(}\alpha_{1}^{\text{OBS}}\text{)}$ as the number of imputations increases (5). Hence, given a large number of imputations, $\text{SE}(\beta_{YX}^{\text{MI}})\approx \text{SE}(\beta_{YX|W})$ when *π*_0_ = 0 and $\text{SE}(\beta_{YX}^{\text{MI}})\to \text{SE}(\beta_{YX|W,R})$ as *π*_0_ → 1 (see Supplementary Material Section S1 for further explanation of this result).

In general, the SE of the OLS estimator of a regression coefficient, SE(*β*), equals the square root of the residual variance divided by the square root of the product of the sample size (*n*) and the variance of *X* for the fitted model. Hence, we can calculate SE(*β*_YX_|_W_) and SE(*β*_YX_|_WR_) as follows: $\text{SE}(\beta_{YX|W})=\sqrt{\frac{\text{Var}(Y-\hat{Y}|X,W)}{n\text{Var}(X|W)}}$ and $\text{SE}(\beta_{YX|W,R})=\sqrt{\frac{\text{Var}(Y-\hat{Y}|X,W,R)}{n\text{Var}(X|W,R)}}$ where, in this setting, *n* represents the number of records with an observed value of *Y*, and $\hat{Y}$ represents the mean value of *Y* predicted using the specified imputation model.

Since Cov(*X, W*) = 0 and Var(*X|W*) - Var(*X*) (see Supplementary Material Section S2 for proof of this and other expressions in this section), SE(*β_YX_|_W_*) can be expressed fairly simply as

(1) $$\sqrt{\frac{\text{Var}(Y)-\beta_{YX}^{2}\text{Var}(X)-{\text{Cov}}^{2}(Y,W)/\text{Var}(W)}{n\text{Var}(X)}}$$

The expression for SE(*β_YX|W,R_*) is more complicated; if the imputation model parameters for *X*, *W* and *R* are denoted by *b*_1_, *b*_2_ and *b*_3_, respectively, SE(*β_YX|W,R_*) has the general form

(2) $$\sqrt{\frac{\begin{array}{l} \text{Var}(Y)-b_{1}^{2}\text{Var}(X|W,R)-b_{2}^{2}\text{Var}(|W|X,R)-b_{3}^{2}\text{Var}(R|X,W)-\\ \left.2b_{1}b_{2}\text{Cov}(X,W|R)-2b_{1}b_{3}\text{Cov}(X,R|W)-2b_{2}b_{3}\text{Cov}|W,R|X\right) \end{array}}{n\text{Var}(X|W,R)}}$$

The size of this expression, relative to the magnitude of Equation 1, will depend on the strength of the associations between *Y*, *X*, *Z*, *W*, *U* and R. Since Var(*X|W, R*) < Var(*X*), if the residual variance (i.e., the numerator in Equation 2) is at least as large as that for SE(*β_YX_|_W_,_R_*) (i.e., the numerator in Equation 1), SE(*β_YX_|_W_,_R_*) will be greater than SE(*β_YX_|_W_*) given the same sample size *n*.

Further note that the SE of the CRA estimator is equal to

(3) $$\text{SE}(\beta_{YX})=\sqrt{\frac{\text{Var}(Y)-\beta_{YX}^{2}\text{Var}(X)}{n\text{Var}(X)}}$$

when *π_0_* = 0, tending to

(4) $$\begin{array}{c} \text{SE}(\beta_{YX|R})=\sqrt{\frac{\text{Var}(Y-\hat{Y}|X,\text{R})}{n\text{Var}(X|\text{R})}}\\ =\sqrt{\frac{\text{Var}(Y)-\beta_{YX}^{2}\text{Var}(X)}{n\{\text{Var}(X)-{\text{Cov}}^{2}(X,R)/\text{Var}(R)\}}} \end{array}$$

as *π*_0_ → 1 (noting *Y* is unrelated to *R* given *X* so $\hat{Y}|X,R=\beta_{YX}X).$ Note this is also, given a large number of imputations, approximately the SE of the MI estimator when only *X* is included in the imputation model. Comparing Equations 3 and 4, we see, as expected, that the SE of the CRA estimator increases as *π*_0_ → 1. Furthermore, comparing Equations 3 and 4 with Equations 1 and 2, the SE of the CRA estimator, or the MI estimator using only *X*, may be greater in magnitude than the SE of the MI estimator including *W* in the imputation model, depending on the strength of the associations between *Y*, *X*, *Z*, *W*, *U*, *R* and *π_0_* (although the SE of the CRA estimator, or the MI estimator using only *X*, will always be greater than the SE of the MI estimator including *W* in the imputation model when *π_0_* = 0, given Cov(*Y, W*) ≠ 0).

### 2.4 Illustration of the bias and standard error of the MI estimator when including a collider in the imputation model for a continuous outcome as the proportion of missing data increases

We illustrate how the bias and SE of the MI estimator when including a collider in the imputation model vary with *π*_0_, using a simple simulation (see Supplementary Material Section S3 for further details). For reference, we also illustrate how the SE of the CRA estimator varies with *π*_0_ (the CRA estimator is always unbiased in this setting). This example is based on the relationships depicted in Figure 1, setting the mean of each variable (including *R*) equal to 0, all direct effect sizes equal to 1 and all error variances equal to 1.

**Figure 2:**
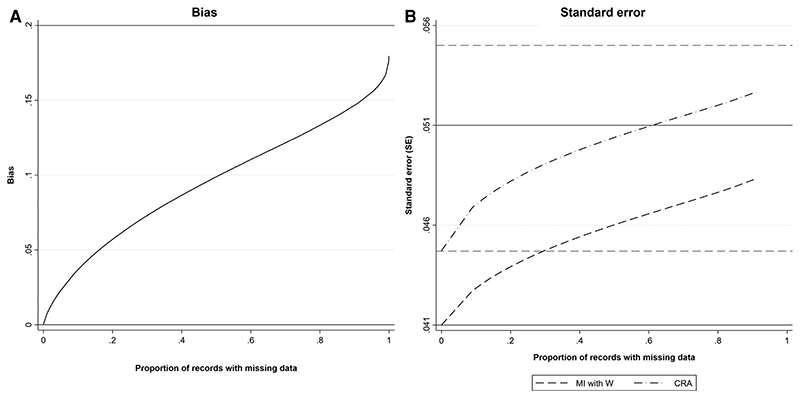
(A) Estimated bias and (B) SE of the MI estimator of *β_YX_* when the imputation model includes a collider, *W*, and SE of the CRA estimator of *β_YX_*, plotted against the proportion of records with missing data, when continuous outcome *Y* is partially observed, assuming 1,000 observed values. All direct effect sizes and error variances equal 1. Horizontal grey solid lines represent the values of bias and SE of the MI estimator when the proportion of records with missing data is 0 (lower line) or tends to 1 (upper line). Horizontal grey dashed lines represent the values of the SE of the CRA estimator when the proportion of records with missing data is 0 (lower line) or tends to 1 (upper line).

Figure 2 shows, as *π_0_* increases, (a) estimated bias and (b) estimates of SE of the MI estimator when the imputation model includes a collider, compared with SE of the CRA estimator. For reference, the true values of *β_YX_*, *β_YX|W,R_*), SE(*β_YX_*), SE(*β_YX|W,R_*), SE(*β_YX_*) and SE(*β*_*YX*|*R*_) are shown (with the residual variance of SE(*β_YX|W,R_*) calculated empirically due to the complexity of the algebraic form for this quantity). As expected, when there were no missing values, bias of the MI estimator equalled 0, SE of the MI estimator was equal to SE(*β_YX|W_*) and SE of the CRA estimator was equal to SE(*β_YX_*). As *π_0_* increased, bias, SE of the MI estimator, and SE of the CRA estimator increased at a similar, approximately linear rate (until *π_0_* was very close to 1), approaching |*β_YX|W,R_* – *β_YX_*|,SE(*β_YX|W,R_*) and SE(*β_YX_|_R_*), respectively, as *π_0_* approached 1. The approximately linear growth with the proportion of missing data is due to the fact that the transformation from the binary indicator *R*_ind_ to the underlying normal variable *R* is approximately linear (16). Bias was approximately half the maximum value when π_0_ = 0.5. In this particular example, for each value of π_0_, the SE of the MI estimator was smaller than the SE of the CRA estimator. However, note that this will not always be the case, e.g., if the strength of the associations between both *Y* and *Z*, and *W* and *Z* are reduced to 0.5 (with the setting otherwise as depicted in Figure 2), the SE of the MI estimator will be greater than the SE of the CRA estimator if the proportion of missing data is greater than approximately 40% (see Supplementary Material Section S1, Figure S1 and also Section S5, Figure S2 which illustrates the relative precision of the MI and CRA estimators for various direct effect sizes). The difference between ${\hat{\beta }}_{YX}^{\text{MI}}\;\text{and}\;{\hat{\alpha }}_{1}^{\text{OBS}}$ was negligible (the median difference was 0.0001, 5th–95th percentile: −0.0003 to 0.0001).

### 2.5 General expression for the maximum bias of the MI estimator when including a collider in the imputation model for a continuous outcome in terms of the direct effect sizes

We next provide a general expression for the maximum bias of the MI estimator when including a collider in the imputation model, in terms of the direct effect sizes and error variances. The maximum bias of the MI estimator when including a collider in the imputation model is

(5) $$\frac{\beta_{RX}\beta_{RU}\beta_{WU}\beta_{YZ}\beta_{WZ}\sigma_{Z}^{2}\sigma_{U}^{2}}{(\beta_{RU}^{2}\sigma_{U}^{2}+\sigma_{R}^{2})(\beta_{WZ}^{2}\sigma_{Z}^{2}+\sigma_{W}^{2})+\beta_{WU}^{2}\sigma_{U}^{2}\sigma_{R}^{2}}$$

where the direct effect sizes are denoted by *β*, e.g., *β_RX_* denotes the direct effect of *X* on *R*, and the variances of the errors are denoted by *σ*^2^, e.g., $\sigma_{X}^{2}$ denotes the variance of the error of X. Equation 5 was verified by simulation (see Supplementary Material Section S4).

From Equation 5, we can see that the magnitude of the maximum bias does not depend on *β*_YX_ and that the direction of the maximum bias depends on the sign of the product *β_RX_β_RU_β_WU_β_YZ_β_WZ_* (because $\frac{\sigma_{Z}^{2}\sigma_{U}^{2}}{(\beta_{WU}^{2}\sigma_{U}^{2}|\sigma_{R}^{2})(\beta_{WZ}^{2}\sigma_{Z}^{2}|\sigma_{W}^{2})+\beta_{WD}^{2}\sigma_{U}^{2}\sigma_{R}^{2}}$ is strictly positive assuming non-zero error variances). There will be no bias if at least one of *β_RX_*, *β_RU_*, *β_WU_*, *β_YZ_* or *β_WZ_* is equal to 0, consistent with the underlying DAG (Figure 1).

### 2.6 Illustration of maximum bias formula for a continuous outcome in terms of the direct effect sizes

We illustrate how the maximum bias varies with the direct effect sizes using a numerical example. In this example, we used moderate values of the direct effect sizes *β*_*RX*_, *β*_*RU*_, *β*_*WU*_, *β*_*YZ*_ and *β*_WZ_ (relative to the error variances $\sigma_{U}^{2},\sigma_{Z}^{2},\sigma_{W}^{2}\;\text{and}\;\sigma_{R}^{2},$ which were all equal to 1): direct effect sizes were each set to 0.00, 0.25, 0.50, 0.75 or 1.00. For *β_RX_* and *β_RU_*, note that these values correspond approximately to odds ratios from a logistic regression model for *R*_ind_ of 1.00, 1.50, 2.30, 3.50 or 5.30 (using the general rule for transforming a parameter from a logistic to a probit model (16) by multiplying the logarithm of the odds ratio by 0.6; note this is valid unless the proportion of complete records is very close to 0 or 1).

**Figure 3:**
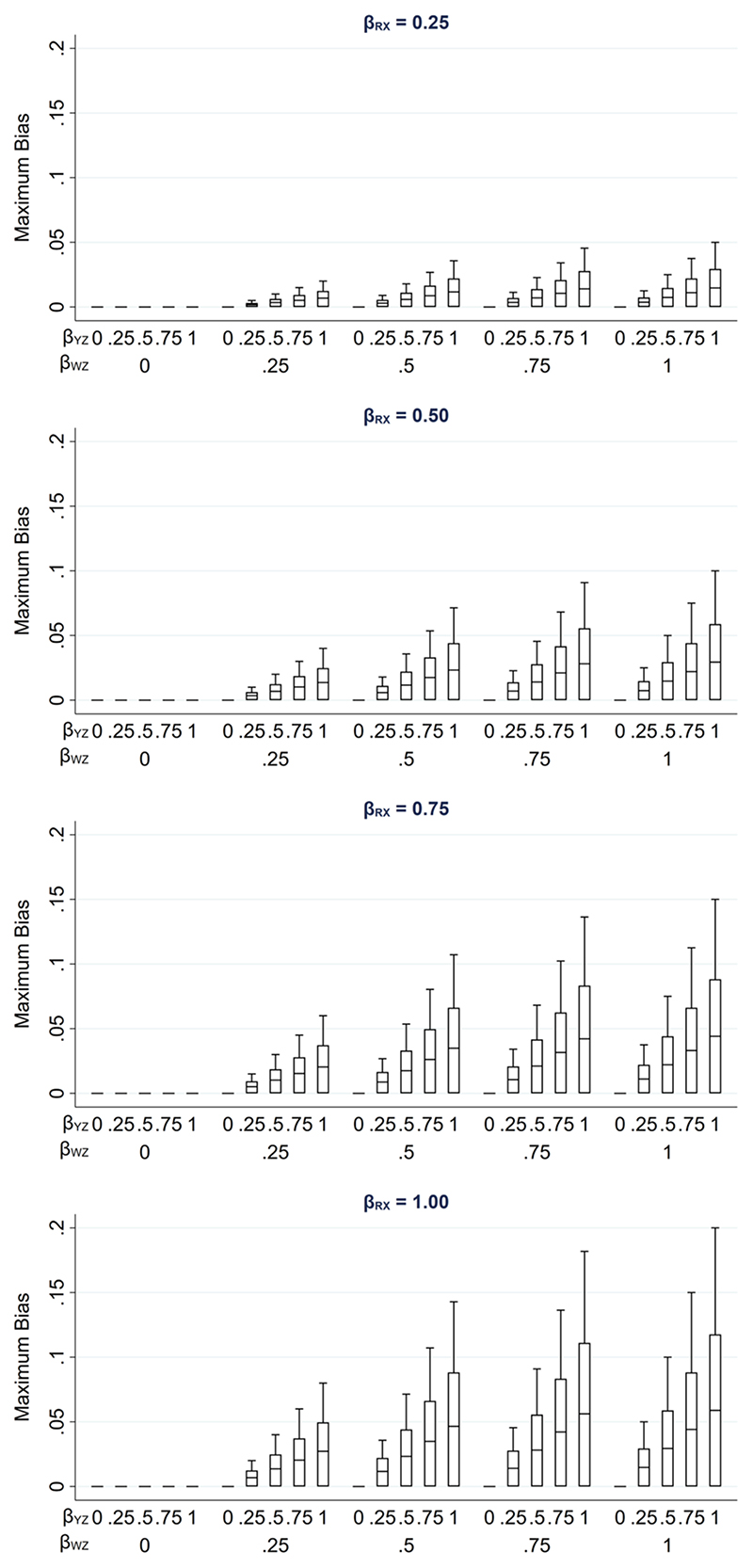
Maximum bias of the MI estimator of *β_YX_* when continuous outcome Y is partially observed, varying direct effect sizes *β_RX_*, *β_RU_*, *β_WU_*, *β_YZ_*> and *β_WZ_*. The distribution of maximum bias in each box plot is averaged over the values of *β_RU_* and *β_WU_*.

Figure 3 illustrates the impact of the direct effect sizes on the maximum bias of the MI estimator. We focus particularly on the impact of *β_RX_*, *β_YZ_* and *β_WZ_* because unbiased estimates of these effect sizes can be calculated using the observed data, assuming that *X*, *W* and *Z* are fully observed and–implicit from Figure 1—that *β_YZ|R_* = *β_YZ_* (note *β_RU_* and *β_WU_* cannot be estimated in our setting because we assume U is unmeasured). In each panel, maximum bias is plotted against *β_YZ_* and *β_WZ_*, for a single value of *β_RX_* (which increases across the panels). The distribution of the maximum bias for each value of *β_RX_*, *β_YZ_* and *β_WZ_* (represented as a box plot) is due to the variation in the other two parameters; that is, each is averaged over the values of *β_RU_* and *β_WU_*.

As noted previously, maximum bias is equal to zero if any of the direct effect sizes are equal to zero (hence the panel with *β_RX_* = 0 is not displayed) and increases take the same parameter values as mentioned above for *β_RU_*, *β_WU_*, *β_YZ_* and *β_WZ_*, but set *β_RX_* to negative values, then the bias would be of the same magnitude but negative.

### 2.7 Relative increase in precision of the MI estimator when including a collider in the imputation model for a continuous outcome in terms of the direct effect sizes

In the setting shown in Figure 1, in which bias was maximised (i.e., as *π*_0_ → 1), we also examined how the relative increase in precision of the MI estimator including *W* in the imputation model, compared with the CRA estimator, varied with the direct effect sizes. All direct effect sizes were set to 0.00, 0.50 or 1.00, and each variable had a mean of zero and an error variance of 1. For each combination of direct effect sizes, the SE of the CRA estimator was calculated algebraically using Equation 4. As above, due to the complexity of the expression for the SE of the MI estimator (Equation 2), this was calculated empirically. The relative increase in precision was calculated as 100 × (1 – (SE of the MI estimator)^2^/(SE of the CRA estimator)^2^). The results are illustrated in Supplementary Material Section S5, Figure S2. As discussed above, these show that, as *π*_0_ → 1, the SE of the MI estimator including *W* in the imputation model can be larger or smaller than the SE of the CRA estimator, depending on the magnitude of the direct effect sizes.

## 3 Bias and SE of the MI estimator including a collider in the imputation model when a continuous exposure is partially observed

### 3.1 Setting in which complete records analysis is valid (missingness of the exposure does not depend on the outcome)

**Figure 4:**
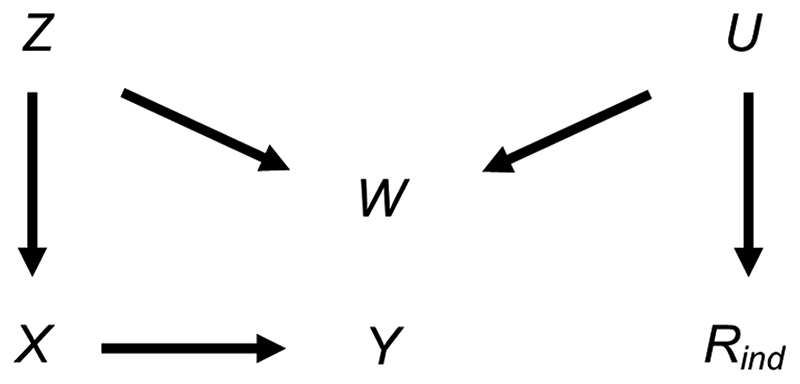
Directed acyclic graph depicting the relationship between outcome *Y*, exposure *X*, missingness indicator *R*_ind_ and potential auxiliary variables *Z*, *W* and *U*. Lines indicate related variables, with arrows indicating the direction of the relationship;absent lines represent variables with no direct causal relation.

We also considered the effect of collider bias in settings in which a continuous exposure *X* was partially observed. First, we considered the setting in which CRA (and MI) was, in principle, valid, with variables related as per Figure 4. In this setting (given the same assumptions and using the same MI method as in the previous setting), the theoretical magnitude of the maximum bias (when including collider *W* in the imputation model for *X*) has a more complicated form because the imputation and substantive models are not the same. Here, the imputation model is of the form: *E*(*X*) = *α*_0_ + *α_1_Y* + *α_2_* W, where *E*(.) denotes the expected value. The MI estimator of *β_YX_* will be unbiased only if an unbiased estimate of each imputation model parameter can be obtained using records with observed values of *X*, i.e., only if $\alpha_{0}^{\text{OBS}}\;\text{and}\;\alpha_{2}^{\text{OBS}}$ and $\alpha_{2}^{\text{OBS}}=\alpha_{2.}$

Taking *α_1_* as an example, and using a similar argument to the previous setting, the bias of $\alpha_{1}^{\text{OBS}}$ is bounded as follows: 0 ≤ bias ≤ |*β*_XY|W,R_ - *β_XY|W_*|. If there are no missing values of *X*, $\alpha_{1}^{\text{OBS}}$ is unbiased. Bias will increase in magnitude with the probability that *X* is missing. In the hypothetical situation in which all values are missing, bias will take its maximum value of |*β*_XY|WR_ – *β_XY|W_*|, where this depends on the magnitude of the conditional and marginal values of both the variance of *Y* and the covariance of *X* and *Y*, as well as the strength of the relationship between *W* and missingness variable *R*; specifically, the maximum bias of $\alpha_{1}^{\text{OBS}\;}=\frac{A\{\text{Var}(Y)\text{Cov}(Y,X|W)-Cav(Y,X)\text{Var}(Y|W)\}}{\text{Var}(Y|W)\{\text{Var}(Y|W)-A\text{Var}(Y)\}},$ where $A=\frac{{\text{Cov}}^{2}(R,W)}{\text{Var}(R)\text{Var}(W)}$ (see Supplementary Material Section S6 for further details of this derivation). Similar expressions can be derived for the maximum bias of $\alpha_{0}^{\text{OBS}}\;\text{and}\;\alpha_{2}^{\text{OBS}}.$

**Figure 5:**
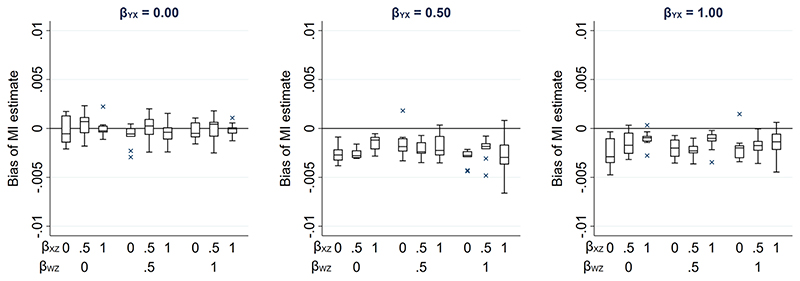
Bias of the MI estimate of *β_YX_*, when 50% of values of a continuous exposure *X* are missing, varying direct effect sizes *β_YX_*, *β_XZ_*, *β_WZ_*, *β_RU_* and *β_WU_*. The distribution of bias in each box plot is averaged over the values of *β_RU_* and *β_WU_*.

Due to its complexity in this setting, an expression for the theoretical magnitude of the maximum bias of the MI estimator is not derived here. However, we illustrate the effect on the MI estimate from including collider *W* in the imputation model by simulation. This example is based on the relationships depicted in Figure 4, setting the mean of each variable (including R) equal to zero, all direct effect sizes equal to 1 and all error variances equal to 1 (see Supplementary Material Section S7 for further details). Note that we refer to the MI or CRA “estimate” when describing simulation study results, rather than “estimator” (which we have used when describing algebraic results). Figure 5 illustrates the impact of the direct effect sizes on the bias of the MI estimate when *X* was missing for 50% of the records, focusing particularly on the impact of *β_YX_*, *β_XZ_ and β_WZ_*. In each panel, bias is plotted against *β_XZ_* and *β_WZ_*, for a single value of *β_YX_* (which increases across the panels). The distribution of the bias for each value of *β_YX_*, *β_XZ_* and *β_WZ_* (represented as a box plot) is due to the variation in the other two parameters; that is, each is averaged over the values of *β_RU_* and *β_WU_*. Figure 5 shows that bias is very small, regardless of the direct effect sizes. In addition, examining the relative increase in precision, compared with the CRA estimate (see Supplementary Material, Section S7, Figure S3), shows that the SE of the MI estimate including W in the imputation model can be larger or smaller than the SE of the CRA estimate, depending on the magnitude of the direct effect sizes.

### 3.2 Setting in which complete records analysis is not valid (missingness of the exposure additionally depends on the outcome)

**Figure 6:**
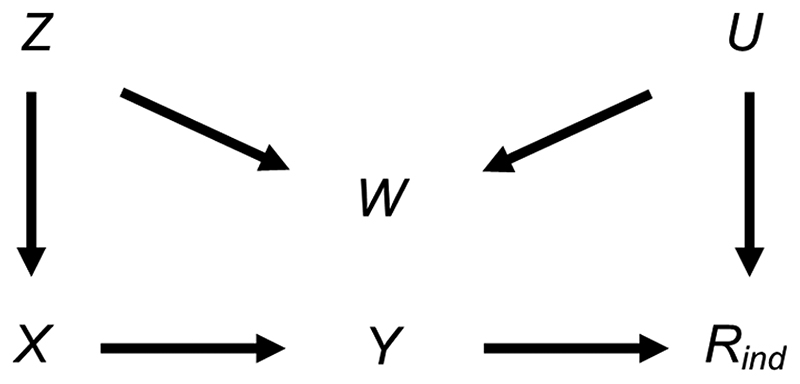
Directed acyclic graph depicting the relationship between outcome *Y*, exposure *X*, missingness indicator *R*_ind_ and potential auxiliary variables *Z*, *W* and *U*. Lines indicate related variables, with arrows indicating the direction of the relationship; absent lines represent variables with no direct causal relation.

**Figure 7:**
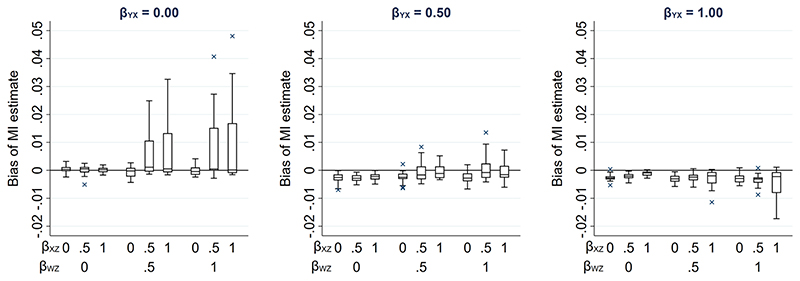
Bias of the MI estimate of *β_YX_* when 50% of values of a continuous exposure *X* are missing and missingness is additionally caused by *Y*, varying direct effect sizes *β_YX_*, *β_XZ_*, *β_WZ_*, *β_RU_*, *β_RY_*, and *β_WU_*. The distribution of bias in each box-plot is averaged over the values of *β_RU_*, *β_RY_*, and *β_WU_*.

In our setting with a partially observed continuous exposure *X*, the magnitude of bias was much smaller than in the setting with a partially observed continuous outcome *Y*. This is because there is only one pathway between the partially observed variable and its missingness in the *X* setting (via *Z-W-U*), whereas there are two pathways in the *Y* setting (via *Z-W-U* and *X*). Hence, the cumulative bias (i.e., the sum of the bias via each pathway) is potentially larger in the *Y* setting. Therefore, to provide a more comparable setting to that when *Y* is partially observed, we considered an additional setting when continuous variable *X* was partially observed, in which *Y* was also a cause of missingness of *X* (Figure 6). The relationships depicted in Figure 6 are the same as those in Figure 4, with the addition of an arrow from *Y* to *R*. There are now two potential pathways between *X* and its missingness, via *Z-W-U* and *Y*. Note that the CRA is no longer valid in this setting, because missingness depends on the analysis outcome *Y*. However, MI using *Y*, or *Y* and *Z*, in the imputation model for *X* would be valid. Using the same simulation approach as before (see Supplementary Material Section S7 for further details), Figure 7 illustrates the effect on the MI estimator from including collider W in the imputation model. Figure 7 shows that when missingness in *X* is caused by *U* and *Y* and *β_YX_* is close to 0, bias is similar in magnitude to that in the setting in which missingness in *Y* is caused by *U* and *X*.

Note that in similar settings to those discussed here, with a binary partially observed variable (i.e., the same settings as depicted in Figures 1, 4 but with either partially observed binary *Y* or partially observed binary *X*), the bias of MI estimates will be approximately the same magnitude as for the continuous cases, provided the probability of each value of the binary variable is not close to 0 or 1 (see Supplementary Material Section S7, Figures S4, S5). This follows in each case by assuming that the binary variable has an underlying normal distribution, in which case the results described here will still approximately apply.

## 4 Real data example

### 4.1 Methods

We illustrate the use of our formula for maximum bias given a partially observed continuous outcome, as per the setting described in Section 2.1, using data from the Avon Longitudinal Study of Parents and Children (ALSPAC). ALSPAC is a prospective study that recruited pregnant women with expected dates of delivery between 1 April 1991 and 31 December 1992, in the Bristol area of the UK (17, 18). We used data from the initial recruitment phase, in which 14,541 pregnant women enrolled, resulting in 14,062 live births (13,988 alive at 1 year of age). Children and their mothers have been followed up since birth through questionnaires, clinics and linkage to routine datasets. Ethical approval for the study was obtained from the ALSPAC Ethics and Law Committee and local research ethics committees. Informed consent for the use of data collected via questionnaires and clinics was obtained from participants following the recommendations of the ALSPAC Ethics and Law Committee at the time.

Here, as described earlier, our substantive model of interest was the regression of child’s BMI at age 7 years (which was partially observed) on maternal education (defined as a binary variable indicating whether the child’s mother held a post-16 years qualification). We restricted analysis to all singletons and first-born twins (excluding the second-born twin to avoid family-level clustering) who were alive at 1 year *(n* = 13,745). For illustrative purposes, as before, we assumed that there were only two candidate auxiliary variables available for use in the imputation model for BMI at age 7 years: pregnancy size (singleton vs. twin birth); and child’s birth weight (in reality, a large amount of individual-level data are available: the ALSPAC study website contains details of all available data through a fully searchable data dictionary and variable search tool: http://www.bristol.ac.uk/alspac/researchers/our-data/). We further assumed that the exposure and auxiliary variables were fully observed (in reality, a small proportion of participants had missing values for these variables: 1,684 participants, 12%, were missing values of maternal education, *n* = 1,510, birth weight, *n* = 150 or both, *n* = 24). Therefore, we analysed 12,061 participants with observed values of maternal education, pregnancy size and birth weight, of whom 7,248 (60%) had an observed value of BMI at age 7 years.

**Figure 8:**
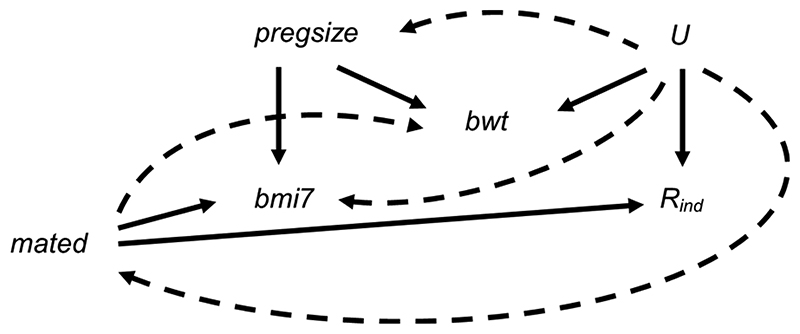
Directed acyclic graph depicting the relationship between child’s body mass index at age 7 years (bmi7), maternal education (mated: a binary variable indicating whether the child’s mother held a post-16 years qualification), pregnancy size (pregsize: singleton or twin birth), child’s birth weight (bwt), missingness indicator *R*_ind_ (a binary variable indicating whether bmi7 is observed) and unobserved variable(s) *U*. Lines indicate related variables, with arrows indicating the direction of the relationship. Straight solid lines depict the relationships assumed in the theoretical scenario in which the analysis model outcome is missing at random; curved dashed lines depict additional relationships that are plausible in our real data example; absent lines represent variables with no direct causal relation.

Figure 8 depicts the relationships between BMI at age 7 years, maternal education, pregnancy size, birth weight and missingness indicator *R*_ind_ (a binary variable indicating whether BMI at age 7 years is observed), plus unmeasured variable(s), *U* [related to the analysis model variables and/or their missingness, e.g., markers of socioeconomic position (SEP)]. Figure 8 shows both the relationships assumed in the theoretical scenario (i.e., as per Figure 1, represented by straight, solid lines) and additional relationships that are plausible in our real data example, based on prior research (19–22) (represented by curved, dashed lines). For example, in the theoretical scenario, we assume that only maternal education and pregnancy size cause BMI at age 7 years, and only maternal education and *U* cause missingness in BMI at age 7 years. In the real data scenario, it is plausible that BMI at age 7 years is MNAR, because *U* may be related to both BMI at age 7 years and *R*_ind_. We assume that pregnancy size is not a cause of *R*_ind_, although pregnancy size may be related to *R*_ind_ via *U* (e.g., because assisted reproduction is associated with higher SEP). Similarly, we assume that birth weight is not a cause of BMI at age 7 years (as per, for example, (23)) or *R*_ind_, but shares a common cause with both, i.e., birth weight is a collider.

We assessed the potential impact on the MI estimate from including a collider (birth weight) in the imputation model for BMI at age 7 years in two steps:

1. We assessed whether our hypothesised relationships with birth weight were plausible by exploring the relationships between maternal education, pregnancy size, birth weight and *R*_ind_. We assessed relationships using linear or logistic regression models (for continuous and binary outcomes, respectively) for each pair of variables in turn (deciding which variable was the dependent variable and which the explanatory variable in any given pair according to the probable causal direction), adjusting for any observed confounders.
2. Based on our results from Step 1, we applied our formula (Equation 5) for maximum bias of the MI estimator if the hypothesised collider birth weight was included in the imputation model for BMI at age 7 years. Since not all the direct effect sizes were estimable from the observed data, we used an alternative (equivalent) version of our maximum bias formula, expressed in terms of the variances and covariances of the observed (or partially observed) variables. We also assumed (without loss of generality) that *R* had a mean of zero and a variance of 1. Therefore, we used the following version of the formula to calculate maximum bias: $\frac{\text{Cov}(X,R)\text{Cov}(W,R)\text{Cov}(Y,W)}{\left\{\text{Var}(X)-{\text{Cov}}^{2}(X,R)\}\text{Var}(W)-\text{Var}(X){\text{Cov}}^{2}(W,R\right)}$

where, in our setting, *X* denotes maternal education, *W* denotes birth weight and *Y* denotes BMI at age 7 years. Since we observe *R*_ind_ (i.e., whether BMI at age 7 years is observed) rather than the underlying normal variable *R*, covariance terms involving *R* were approximated by applying the general rule for transforming a parameter from a logistic to a probit model (16), as before, such that: Cov(., *R*) = 0.6 × logOR_*R_ind_*_ ×Var(.), where logOR*R*_ind_ denotes the logarithm of the odds ratio (i.e., the regression parameter) from a logistic regression of *R*_ind_ on the specified covariate. For example, Cov(*X*, *R*) was approximated by 0.6 × logOR_*R_indX_*_ ×Var(X). We estimated Var(X) using the normal approximation to the binomial because *X* was binary. We estimated Cov(Y, W) using the complete records and other terms using all records. For simplicity, we assumed that the relationship between birth weight and BMI at age 7 years was linear. We also assumed that estimates of the variances and covariances used in our maximum bias formula were unbiased (which may not have been the case if *Y* was MNAR or if there were unmeasured confounders).

We compared our estimate of the exposure coefficient based on our formula for maximum bias to both the CRA estimate and MI estimates using no auxiliary variables or either pregnancy size, birth weight or both, as auxiliary variables. Each imputation model also included the analysis model exposure, maternal education. We used a large number of imputations (100) to ensure we obtained stable estimates of the exposure coefficient and its SE.

### 4.2 Results: magnitude of bias due to a collider auxiliary variable

#### 4.2.1 Step 1

Relationships between maternal education, pregnancy size, birth weight and *R*_ind_ are summarised in Table 2. In particular, these suggest that *R*_ind_ is strongly associated with both maternal education and birth weight, but less so with pregnancy size. However, adjusting for birth weight increases the strength of the relationship between *R*_ind_ and pregnancy size [unadjusted odds ratio (OR): 1.07, 95% confidence interval (CI): 0.77–1.48 vs. adjusted OR: 1.25, 95% CI: 0.90–1.75]. These results, combined with our prior knowledge of the data, suggest that birth weight is a collider. Therefore, inclusion of birth weight in the imputation model for BMI at age 7 years may induce or inflate bias due to data being MNAR.

#### 4.2.2 Step 2

Substituting values based on the observed data [as per Table 2, and additionally, using estimates of Var(*W*), Var(*X*) and Cov(*Y, W*) of 0.286, 0.228 and 0.171, respectively] into our theoretical expression, we estimated the maximum bias from including birth weight in the imputation model for BMI at age 7 years to be 0.008 (towards the null). We can use the SE of the MI estimate including birth weight (Table 3) as an approximate estimate of the SE of the maximum bias (assuming the true value of the exposure coefficient is fixed, i.e., does not vary). Thus, we can construct an approximate confidence interval for the maximum bias as follows: maximum bias ± 1.96 × SE of the MI estimate including birth weight. This gives a 95% CI of −0.084 to 0.100, although we note there may be additional variation due to uncertainty about the estimated effect sizes and variance/covariance terms used in our formula. This result suggests that even though there is the possibility of collider bias due to inclusion of birth weight in the imputation model, the magnitude of bias is small in this particular setting.

The analysis results (Table 3) confirmed that the CRA and MI estimates of the exposure coefficient were very similar, regardless of the auxiliary variable(s) used in the MI procedure. However, as predicted, there was slight attenuation in the MI estimate when birth weight was included in the imputation model for BMI at age 7 years. This was the case even when pregnancy size was also included. This suggests that there was at least one other unobserved variable that had similar relationships with other variables as pregnancy size (e.g., child’s sex), so adjusting for pregnancy size did not completely remove the bias induced by inclusion of birth weight in the imputation model. The difference between the CRA estimate and the MI estimate including birth weight was 0.023 (towards the null), which was larger than our estimate based on the theoretical magnitude of bias, although in the same direction and within the approximate confidence interval.

As expected, the SE of the CRA estimate was similar to the SE of the MI estimate using no auxiliary variables and larger than the SE for MI estimates using pregnancy size or birth weight as auxiliary variables. However, the SE of the MI estimate using both pregnancy size and birth weight as auxiliary variables was larger than that for all other analysis strategies. This may be because pregnancy size has only a weak direct effect on BMI at age 7 years, i.e., pregnancy size is largely redundant if the imputation model already includes birth weight; thus, its addition leads to a decrease in precision (5).

## 5 Discussion

In this paper, we quantify, algebraically and by simulation, the magnitude of bias and SE of the MI estimator induced by including a collider in the imputation model, in settings where it is possible to specify an imputation model that gives unbiased inference for the population parameter values. We have derived an algebraic expression for the maximum bias and its relationship to the proportion of incomplete records when a continuous outcome is partially observed. We have demonstrated that in this setting (and also if the outcome is binary), the bias can be substantial, relative to the magnitude of the exposure coefficient. We found, in settings in which the CRA was valid, the bias due to the inclusion of a collider in the imputation model was smaller when the exposure in the analysis model (either binary or continuous) was partially observed. However, bias was larger in magnitude if the outcome also caused missingness in the exposure (in which case the CRA was no longer valid but MI, using a correctly specified imputation model and correct choice of auxiliary variables, was valid).

When the outcome is partially observed, we have shown that the magnitude of the bias of the MI estimator from including a collider in the imputation model depends on the magnitude of the associations between the exposure and missingness, between the collider and missingness, and between the collider and the outcome, as well as on the proportion of missing data. Crucially, it does not depend on the magnitude of the association between outcome and exposure. Therefore, if the association between outcome and exposure is much weaker than the associations between other pairs of variables and the proportion of incomplete records is fairly large (precisely the situation in which one may wish to use auxiliary variables), the relative bias of the MI estimator could be substantial.

In our real data example, we assumed that both auxiliary variables (direct predictor pregnancy size and collider birth weight) were measured. However, note that the bias can still be estimated even if the direct predictor is unmeasured, because the maximum bias formula does not depend on this variable. However, in this case, assessing whether an auxiliary variable is a collider may need to rely on both prior knowledge and inspection of the hypothetical causal model of interest, because it may be difficult to assess whether it is a collider using the observed data alone. The likely impact of including a collider in the imputation model(s) can still be assessed using our suggested formula and/or our plots based on simulations, estimating the strength of each relevant association using either the observed data or published results. In our theoretical settings, the MI estimator is unbiased when *Z* is included in the imputation model, in addition to *W* (implicit from the DAG for each setting). However, in practice, this strategy may still result in biased estimates, due to unmeasured confounding of the relationship between *Y* and *W*. For example, in our real data analysis, adjusting for pregnancy size did not remove the bias induced by inclusion of the collider, birth weight, in the imputation model due to unmeasured confounding of the relationship between BMI at age 7 years and birth weight. Therefore, we recommend that a collider should not be included in the imputation model (as opposed to including a collider and then attempting to mitigate for its inclusion using other auxiliary variables).

In addition to inducing bias, including a collider in the imputation model may increase, rather than decrease, the SE of the MI estimator. We have shown that this depends on the magnitude of the associations between the exposure, outcome, collider and missingness. However, inclusion of a collider in the imputation model may recover more information about the missing data than CRA, or MI including only the other analysis model variables in the imputation model, and increase precision. Therefore, where the likely bias from inclusion of a collider is small, we recommend performing a sensitivity analysis, comparing the precision of the MI estimate when the imputation model does or does not include a collider. If the gain in precision is sufficiently large, it may be preferable to include a collider in the imputation model, at the expense of some bias, especially if no other auxiliary variables are available. Conversely, if both the gain in precision *and* the likely bias are large (noting that the direction of bias depends on the signs of the associations between other pairs of variables and not on the sign of the association between outcome and exposure), the inclusion of a collider could lead to more precise, but incorrect, estimates of both the strength and direction of the effect. It is possible, for example, that this could result in a weak positive association being incorrectly estimated as a strong negative association.

A strength of our approach is that we have considered a range of commonly occurring scenarios, in which the partially observed variable is either the analysis model outcome or the exposure, as well as either continuous or binary. By using both algebraic quantification and simulation, we have been able to provide a detailed illustration of the effect on both bias and SE, and how these are related to the magnitude and sign of individual associations between exposure, outcome, auxiliary variables and missingness. A limitation is that we have only considered simple models in which variables are normally distributed, or binary, without interactions or non-linear relationships. Our results for the magnitude of bias and SE naturally extend to certain types of skewed/non-normal and categorical variables, e.g., a variable with a log-normal distribution, or a polytomous variable that can be expressed as a set of binary “dummy” variables. In addition, because our general argument is based on the DAG for the substantive model of interest, which does not make any distributional assumptions, our findings also extend to more complex situations, e.g., if there is an exposure–confounder interaction. In this case, the expression for the maximum bias would be more complicated (and the relationship between maximum bias and the direct effect sizes may be non-linear). In applied examples with specific forms for the variables and their relationships, simulation could be used to assess the likely magnitude of the bias and SE if a collider is included in the imputation model. Furthermore, although we have only considered settings in which the MAR assumption was valid, note that even if data were MNAR (in which case the MI estimator would be biased), inclusion of a collider as an auxiliary variable could amplify this bias (7).

A further limitation of our study is that in each of our scenarios, only a single variable has missing values. When multiple variables have missing values, assessing whether imputation models include colliders is likely to be a more complex process. If multiple missingness is handled using MI by chained equations (also known as MI by fully conditional specification) (24), each imputation model only considers one variable to have missing values, as here. In this case, auxiliary variables should be considered separately for each imputation model, because an auxiliary variable may be a collider for one partially observed variable, but not another. If an auxiliary variable is included in several imputation models and could be acollider in all of them, then the bias may be amplified across the imputed variables. For example, returning to our real data setting, if our exposure was also partially observed, and we included birth weight in the imputation model for both our outcome, BMI at age 7 years, and our exposure, maternal education (noting that the default in most software implementations of MI is to include all the listed predictors in the imputation models for all partially observed variables), we may expect more bias than when just including birth weight in the imputation model for our outcome, BMI at age 7 years.

In summary, we conclude that, although auxiliary variables have the potential to improve precision of the MI estimate and reduce bias compared with an imputation model that only includes analysis model variables, poorly chosen auxiliary variables can increase both bias and SE. Therefore, it is important that auxiliary variables are selected carefully. In particular, we recommend examining whether any potential auxiliary variables are colliders. This can be achieved through a combination of data exploration and consideration of the plausible casual diagrams and missingness mechanisms (e.g., by using a missingness DAG (25, 26)).

## Supplementary Material

Supplementary Material

## Figures and Tables

**Table 1:** Missing data definitions.

Term	Definition
CRA	Analysis is restricted to individuals who have complete data for all variables in the analysis model.
MCAR	The probability that data are missing is independent of the observed and missing values of variables in the analysis model, and of any related variables. Data can be MCAR if missingness is caused by a variable independent of all these, e.g., if missingness is for administrative reasons.
MAR	Given the observed data, the probability that data are missing is independent of the true values of the incomplete variable. Any systematic differences between the observed and missing values can be explained by associations with the observed data.
MNAR	If data are neither MCAR nor MAR, data are said to be MNAR. The probability that data are missing depends on the (unobserved) values of the incomplete variable, even after conditioning on the observed data.
MI	MI is a method for handling missing data. It consists of three steps: 1. An imputation model is fitted to the observed data (this is usually some form of regression model). The missing values are replaced with draws (“imputed”) from their conditional predictive distribution (after first perturbing the model parameters). This imputation stage is carried out multiple (M) times, to give M completed datasets. 2. The analysis model is fitted to each of the M completed datasets. 3. The M sets of results are combined using Rubin’s rules (2), to correctly account for the uncertainty about the missing values.
Auxiliary variable	A variable that is not in the analysis model but that is included as a predictor in the imputation model to recover information about the missing data.

**Table 2:** Relationships between maternal education (mated), pregnancy size (pregsize), child’s birth weight (bwt) and whether child’s BMI was observed at age 7 years (*R*_ind_), determined using linear or logistic regression models (for continuous and binary outcomes, respectively).

		Dependent variable			
		pregsize	bwt	*R*_ind_
Explanatory variable	mated	Odds of twin birth is slightly reduced when mother holds a post-16 years qualification (OR: 0.96, 95% CI: 0.69-1.34)	Mean birth weight increases by 0.05 kg (95% CI: 0.03-0.07) when mother holds a post-16 years qualification	Odds of observed BMI at 7 years is twice as great when mother holds a post-16 years qualification (OR: 2.31, 95% CI: 2.13-2.51)
	pregsize		Mean birth weight decreases by 0.91 kg (95% CI: 0.82-0.99) for twin birth (vs. singleton)	Odds of observed BMI at 7 years is slightly greater for a twin birth (vs. singleton) (OR: 1.07, 95% CI: 0.77-1.48). **Conditional on birth weight, relationship appears stronger (OR: 1.25, 95% CI: 0.90**-**1.75)**
	bwt			Conditional on maternal education, odds of observed BMI at 7 years increases for each kg increase in birth weight (OR: 1.15, 95% CI: 1.07-1.23)
	Unmeasured variable(s)	Possibly related—cannot be assessed using the observed data		

For each cell, the row indicates the explanatory variable and the column indicates the dependent variable of the regression model. All parameter values are estimates based on the full data and are conditional on any observed confounders. Relationships opposite to the probable direction of causality were not assessed. We assume that maternal education is not caused by any other observed variable, and that whether BMI is observed at age 7 years is not a cause of any other variable. We note that, in addition to the observed relationships depicted, each observed variable may be related to other, unmeasured variable(s). The bold text emphasises the key result in this table.

**Table 3:** Mean change in child’s body mass index (kg/m^2^) at age 7 years when mother holds a post-16 years qualification (vs. no post-16 years qualification), estimated using different analysis strategies.

Analysis strategy	Estimate (SE)	95% CI
Complete records analysis	−0.108 (0.049)	−0.203 to −0.013
MI with no auxiliary variables	−0.106 (0.049)	−0.209 to −0.011
MI with pregnancy size as auxiliary variable	−0.107 (0.047)	−0.198 to −0.015
MI with child’s birth weight as auxiliary variable	−0.085 (0.047)	−0.176 to 0.007
MI with pregnancy size and child’s birth weight as auxiliary variables	−0.091 (0.050)	−0.189 to 0.006

## Data Availability

The data analysed in this study are subject to the following licenses/restrictions: the Stata code to verify theoretical results, and also to generate and analyse the data as per the simulation studies is included in Supplementary Material, Section S8. The Stata code to analyse the real data example is included in Supplementary Material, Section S9. The real data are not publicly available due to privacy restrictions. Requests to access these datasets should be directed to alspac-data@bristol.ac.uk.
